# Decoding Roles of Exosomal lncRNAs in Tumor-Immune Regulation and Therapeutic Potential

**DOI:** 10.3390/cancers15010286

**Published:** 2022-12-31

**Authors:** Wenqin Zhang, Yuanliang Yan, Jinwu Peng, Abhimanyu Thakur, Ning Bai, Keda Yang, Zhijie Xu

**Affiliations:** 1 Department of Pathology, Xiangya Changde Hospital, Changde 415000, China; 2 Department of Pharmacy, Xiangya Hospital, Central South University, Changsha 410008, China; 3 Department of Pathology, Xiangya Hospital, Central South University, Changsha 410008, China; 4 National Clinical Research Center for Geriatric Disorders, Xiangya Hospital, Central South University, Changsha 410008, China; 5 Ben May Department for Cancer Research, Pritzker School of Molecular Engineering, University of Chicago, Chicago, IL 60637, USA; 6 Department of General Surgery, Xiangya Hospital, Central South University, Changsha 410008, China

**Keywords:** cancer, exosomes, lncRNA, immunoregulation, immunotherapy

## Abstract

**Simple Summary:**

The tumor microenvironment (TME) exhibits a pivotal function in the progression of cancer through the bidirectional communication between cellular and noncellular components. During the exchange and interplay of biological signals between immune cells and tumor cells in TME, exosomal lncRNAs are viewed as one of the vital factors, which can interfere with innate and adaptive immune responses to affect the therapeutic efficiency. This review aims to summarize the advance in the roles of exosomal lncRNAs on tumor-immune regulation and propose potential treatment strategies.

**Abstract:**

Exosomes are nanovesicles secreted into biofluids by various cell types and have been implicated in different physiological and pathological processes. Interestingly, a plethora of studies emphasized the mediating role of exosomes in the bidirectional communication between donor and recipient cells. Among the various cargoes of exosomes, long non-coding RNAs (lncRNAs) have been identified as crucial regulators between cancer cells and immune cells in the tumor microenvironment (TME) that can interfere with innate and adaptive immune responses to affect the therapeutic efficiency. Recently, a few major studies have focused on the exosomal lncRNA-mediated interaction between cancer cells and immune cells infiltrated into TME. Nevertheless, a dearth of studies pertains to the immune regulating role of exosomal lncRNAs in cancer and is still in the early stages. Comprehensive mechanisms of exosomal lncRNAs in tumor immunity are not well understood. Herein, we provide an overview of the immunomodulatory function of exosomal lncRNAs in cancer and treatment resistance. In addition, we also summarize the potential therapeutic strategies toward exosomal lncRNAs in TME.

## 1. Background

Cancer is still life-threatening, with sustained growth in incidence and mortality worldwide. In tumor occurrence and progression, the tumor microenvironment (TME) has been recognized as a complex and diverse multicellular ecosystem shaped by neoplastic cells [1]. Constituted by cellular agents (tumor cells, immune cells, endothelial cells, fibroblasts, etc.) and other soluble mediators (cytokines, chemokines, extracellular vesicles, etc.) [2,3], TME exhibits a pivotal function in the progression of cancer through the bidirectional communication between cellular and noncellular components. Noteworthy, the transmission of stimulatory signals between tumor cells and immune cells in TME contributes to tumor progression by evading immune surveillance [4,5]. Accordingly, disruption of the signal delivery has drawn much research interest and could prove a potential intervention target in cancer. A growing body of studies has shown that cargo-carrying extracellular vesicles (EVs) are critical messengers in the interaction between tumor cells and the microenvironment [6,7]. In addition, non-coding RNAs (ncRNAs), such as long-noncoding RNAs (lncRNAs), have been implicated in abnormal immune responses and the subsequent tumor progression and therapy resistance [8,9]. Moreover, lncRNAs can be encapsulated by EVs and delivered to recipient cells in TME [10]. Therefore, understanding the mechanism of the dysregulated immune response involved in TME, which is mediated by the transportation of lncRNAs through EVs, is critical for identifying the possible therapeutic approach to cancer. 

EVs are lipid bilayer-bounded vesicles originating from various cells, such as tumor cells, stromal cells, and immune cells, and diffusely present in a wide range of biofluids such as blood, sweat, urine, and ascites [11,12]. EVs are further classified into exosomes, micro-vesicles, and apoptotic bodies based on their sizes and biogenesis mechanisms [13]. In exosome production and secretion, cellular membranes tend to invaginate to form the early endosomes. Thereafter, the early endosomes are transformed into late endosomes by the Golgi complex. The late endosomal compartments limiting membranes further invaginate to form intraluminal vesicles (ILVs) while encapsulating cargoes from donor cells. Plenty of ILVs aggregate into multivesicular bodies (MVBs). Some MVBs are degraded by intracellular lysosomes, and a few can merge with the cytoplasmic membrane, thus releasing ILVs to the extracellular environment, ultimately forming exosomes [14]. According to the newly-revised ISEV2018 guideline, the naming standard of EVs is based on the physical characteristics (size or density), biochemical composition, or cell of origin [15]. In this review, all cited studies used exosomes sedimented at 100.000 g or defined as exosomes based on their protein composition and identified by transmission electron microscope. 

Since exosomes can be loaded with biological information, including proteins and nuclear acids (e.g., messenger RNAs (mRNAs), microRNAs (miRNAs), and lncRNAs) [16], they are widely known as shuttles for cell-cell communication, usually along with the transmission of biological signals [17]. Such exosome-mediated signal exchanges between cells exist in physiological conditions, such as embryonic development and material metabolism [18], and also participate in tumor progression. An increasing number of studies have indicated that exosomes may exert a key function in immune regulation and thus contribute to establishing an immunosuppressive TME [19]. For example, CD73 expression of exosomes in the serum of melanoma patients can inhibit the immune response of T cells, thus enhancing the resistance to immunotherapy [20]. Melanoma-derived exosomes can inhibit the infiltration of CD8^+^T cells into tumors [21]. Pancreatic cancer-secreted exosomes can boost the conversion of tumor-associated macrophages to M2 phenotype to favor tumor metastasis [22]. Colorectal cancer-derived exosomes induced the expansion of regulatory T cells (Tregs) to construct an immunosuppressive TME that promotes tumor growth and chemoresistance [23]. Taken together, these studies have demonstrated that exosomes may regulate the innate and adaptive immune cells in TME to affect tumor progression and treatment. Accordingly, systematically clarifying the mechanism of exosomes targeting the immune response of cancer may be beneficial to explore potential therapeutic options.

In the human genome, no more than 2% of genes can be transcribed into mRNAs and thus translated into proteins [24]. However, most genes are transcribed into RNAs without the protein-coding ability known as ncRNAs [24]. According to the length of nucleotides, ncRNAs are mainly classified into (1) short ncRNAs, including miRNAs, small interfering RNAs (siRNAs), and small nucleolar RNAs (snoRNAs), and (2) lncRNAs [25]. With the development of sequencing technology, much more quantities of lncRNAs have been found in the human genome. Furthermore, lncRNAs can affect gene expression through more complex mechanisms by interplaying with DNA, RNA, and protein [26]. Therefore, lncRNAs have rapidly become the research focus in recent years. 

**Figure 1:**
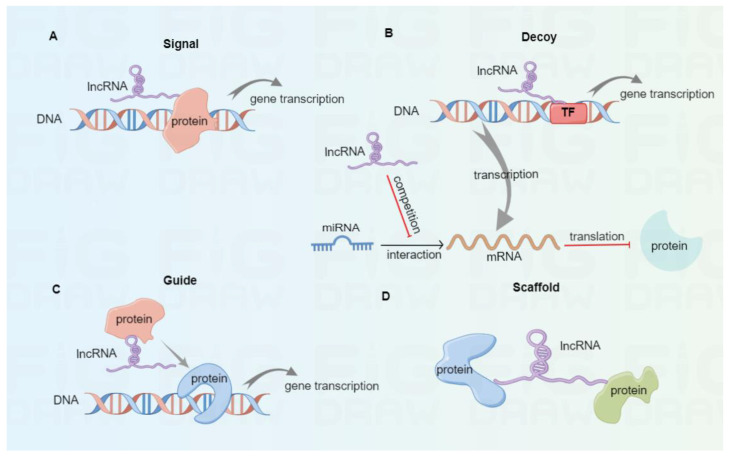
The classification of lncRNAs based on molecular mechanisms. (**A**) Signal lncRNAs can modulate downstream gene transcription independently or by interacting with proteins. (**B**) Decoy lncRNAs serve as decoys of transcription factors or ceRNAs to bind with miRNAs, thus eliminating their inhibitory effect in mRNA translation. (**C**) Guide lncRNAs can bring protein complexes to target sites to promote gene transcription. (**D**) Scaffold lncRNAs serve as scaffold molecules to interplay with multiple protein complexes.

As a class of ncRNAs, lncRNAs involve more than 200 nucleotides and exist in the nucleus and cytoplasm [27]. Based on the transcription onset and direction relative to neighboring protein-coding genes, lncRNAs are classified into sense, antisense, intronic, bidirectional, and intergenic [28]. According to their molecular mechanisms (Figure 1), lncRNAs are divided into signal, decoy, guide, and scaffold [29,30]. Once subjected to external stimulation, signal lncRNAs can modulate the downstream gene transcription independently or interact with proteins like transcription factors, thus affecting specific signaling pathways. For instance, the p53-induced lncRNA-p21 was found to interplay with the heterogeneous nuclear ribonucleoprotein-K, thus suppressing the expression of subsequent genes related to the p53 signaling pathway [31]. Decoy lncRNAs function as decoys of transcription factors or competing endogenous RNAs (ceRNAs) to trap miRNAs and reverse their inhibitory effect in mRNA translation. In tongue squamous cell carcinoma, lncKRT16P6 acts as a trap for binding miR-3180 to promote the expression of GATA zinc finger domain containing 2A (GATAD2A), thus leading to tumorigenesis and metastasis [32]. Highly expressed lncRNA MALAT1 promotes tumor cell migration and invasion by sinking miR-384 in glioma [33]. Guide lncRNAs can bring ribonucleoprotein complexes, such as chromatin modification enzymes, to target positions for various biological functions. Xiao et al. reported that LINC00839 boosted the formation of RUVBL1/Tip60 complexes and guided them to the promoter of NRF1 to induce NRF1 expression, therefore affecting colorectal cancer progression [34]. Scaffold lncRNAs can serve as platforms to interplay with multiple protein complexes, thus facilitating the fusion and incorporation of messages among diverse signaling pathways. For instance, lncRNA ENSG00000274093.1 was found to promote the combination of histone deacetylase 2 (HDAC2) with histone methyltransferase EZH2 to induce epithelial-mesenchymal transition, thus promoting the migration and invasion of colorectal cancer cells [35]. 

Owing to their transcriptional modulation to DNA, post-transcriptional modulation to target miRNA, and epigenetic regulation to proteins, lncRNAs are involved in many physiological processes, such as cell survival, growth [36,37], and maintenance of stemness [38]. Accumulating studies have demonstrated that dysregulated expression of lncRNAs is closely associated with tumor progression and metastasis [39]. Notably, lncRNAs can modulate tumor immunity between tumor cells and immune cells in TME. For instance, Fang et al. demonstrated that lncRNA GAS5 overexpression in NK cells increases their cytotoxicity to liver cancer [40]. Upregulated LncRNA HCG18 in tumor tissues was reported to inhibit CD8^+^T cells, thus increasing the chemoresistance of colorectal cancer [41]. LncRNA-PACERR was found to promote the polarization of M2 macrophages to regulate the progression of pancreatic ductal adenocarcinoma [42]. These results suggest that lncRNAs can participate in tumor progression by regulating the function of immune cells. Therefore, further understanding the role of lncRNAs in regulatory tumor immunity can provide insights for tumor immunotherapy.

During the exchange and interplay of biological signals between non-tumor cells and tumor cells in TME, exosomal lncRNAs are viewed as one of the vital factors affecting tumorigenesis and progression. Since many kinds of cells infiltrated into TME, several stromal cells, such as CAFs and endothelial cells, also communicate with tumor cells based on exosomal lncRNAs. CAFs can promote immune suppression by inhibiting the function of immune cells or secreting inflammatory factors in TME [43]. Inflammatory cytokines, such as IL-6, can take part in the regulation of tumor activity [44]. For instance, LncRNA POU3F3 can be packaged into exosomes derived from esophageal squamous cell carcinoma cells (ESCC) to promote the transformation from normal fibroblasts (NFs) into CAFs. The increased CAFs can enhance the cisplatin resistance of ESCC by producing IL-6 [45]. Ding et al. discovered an uncharacterized lncRNA FLJ22447, which can be transported to fibroblasts via tumor-derived exosomes. High expression of lncRNA FLJ22447 was found to promote the transformation of NFs to CAFs by upregulating IL-33 expression, thus facilitating OSCC progression [46]. In addition, CD90^+^ liver cancer cell-secreted exosomes containing lncRNA H19 can be transferred to endothelial cells to promote angiogenesis and increase their adhesive characteristic, thereby leading to tumor metastasis [47]. These results indicate that exosomal lncRNAs also mediate the communication between stromal cells and tumor cells to affect tumor progression. Accordingly, searching for potential therapeutics to target exosomal lncRNAs may offer value in cancer treatment.

In addition, immune cells infiltrating into TME mainly include tumor-associated macrophages (TAMs), neutrophils, NK cells, and T and B cell subsets [48]. These immune cells could interact with tumor cells to exert pro- or anti-tumor effects, which may be attributed to exosomal lncRNAs mediated immune regulation in TME [49,50]. Although the effects of exosomes or lncRNAs alone in cancer immunity are still under investigation, the immunomodulation effects of exosome-derived lncRNAs in cancer also need clarification. In this review, we used the combined terms “exosomes,” “lncRNAs,” and “cancer immune” to search the related literature in the PubMed database and summarized the newest advance in the roles of exosomal lncRNAs on tumor-immune regulation and potential treatment strategies.

## 2. Exosomal lncRNA-Mediated Immune Regulation

The immune responses of the host are classically divided into innate and adaptive immunity against foreign substances, which are known as antigens. Innate immunity (also called natural immunity) tends to occur early and is the first barrier against microorganism invasion. It primarily comprises physical barriers like epithelial surface, cells (neutrophils, macrophages, natural killer (NK) cells, dendritic cells, etc.), and noncellular factors (complements and mediators of inflammation) [51,52]. Intact epithelial barriers construct the first line of defense to impede microbial entry into the host. The macrophages/neutrophils-mediated phagocytosis or the NK cells-mediated killing activity could eliminate the microbes and infected cells inside the host [53]. In addition, innate immunity could initiate adaptive immunity through the secretion of cytokines or complement fragments. Adaptive immunity (acquired immunity) is often divided into B-cell-mediated humoral immunity and T-cell-mediated cellular immunity [54]. In humoral immunity, activated B cells secrete antibodies to eliminate extracellular microbes. After recognizing the antigens presented by dendritic cells, naive CD4^+^T cells can be activated and differentiated into effector CD4^+^T cells. Moreover, naive CD8^+^T cells can be activated and developed into CD8^+^ cytotoxic T lymphocytes (CTLs) targeting phagocytosed and intracellular microbes in adaptive immunity. As one of the classical immune response mechanisms, effector CD4^+^T cells secrete cytokines to activate macrophages to eliminate microbes. Meanwhile, effector CD8^+^T cell-mediated cytotoxicity produces granzymes and perforins to cause apoptotic death of the infected cells [55]. In addition, the naive T and B cells could also differentiate into long-lived memory T and B cells during the microorganism stimulation. Once exposed to the same microbial stimulation, the immune system can mobilize memory T and B cells to respond more rapidly and vigorously, known as immunological memory [56,57]. Based on the current study results, we discussed the regulation role of exosomal lncRNAs secreted from tumor cells or immune cells in innate or adaptive immunity.

### 2.1. Tumor Cell-Derived Exosomal lncRNAs 

#### 2.1.1. The Role of Exosomal lncRNAs in Macrophages

Macrophages in TME are also called tumor-associated macrophages (TAMs) and are a crucial population among innate immune cells [58]. TAMs play pro-tumor or anti-tumor roles depending on their activation status. After exposure to diverse stimuli, TAMs can be transformed into classical M1 or alternative M2 macrophages. M1 macrophages are more likely to produce pro-inflammatory cytokines and possess anti-tumor immunity, whereas M2 macrophages are responsible for anti-inflammation and pro-tumor progression [59]. The conversion of TAMs to M1 or M2 macrophages often occurs in the tumor progression from the early to the advanced stage [60]. Therefore, the interaction between TAM and cancer cells through exosomes needs investigation. 

Brain cancer. Microglia are the unique lineage of resident macrophages in the brain [61]. Once activated, microglia can be polarized to M1 or M2 phenotypes. Xing et al. found that the downregulation of lncRNA XIST promoted the production of exosomes containing miRNA-503 in brain metastasis tumors of breast cancer, converting microglia to M2 to exert tumor-promoting effects [62]. In addition, exosomal lncRNA TALC secreted by glioblastoma can be transmitted to microglia, producing complement C5/C5a to activate the ENO1/p38 MAPK axis, thus manipulating the M2 polarization to achieve temozolomide resistance [63].

Colorectal cancer. Transcriptomics analysis has revealed the upregulation of 13 lncRNAs in BRAF mutant colorectal cancer (CRC) cells. The 13 lncRNAs can be secreted to promote the CD163^+^M2 macrophage polarization through exosomes. The increased IL-6 production from M2 and the elevated TGF-β generation from cancer-associated fibroblasts (CAFs) constitute the pro-tumor immunosuppressive microenvironment [64]. Furthermore, exosomal lncRNA RPPH1 can be transported to macrophages to provoke M2 polarization, thus promoting CRC cell metastasis and proliferation indicated by the increased levels of vimentin and Ki-67 [65]. Furthermore, exosomal lnc-HOXB8-1:2 secreted from neuroendocrine differentiation CRC cells is also found to promote M2 polarization, thus facilitating the progression and metastasis of CRC cells [66].

Esophageal and gastric cancer. Esophageal cancer (EC) cell-secreted exosomal lncRNA RP11-465B22.8 could be transferred to macrophages to induce M2 polarization, resulting in tumor cell migration and invasion [67]. Gastric cancer (GC) cell-secreted exosomal lncRNA HCG18 can promote the polarization of M2 macrophages by lessening miR-875-3p to enhance the expression of Kruppel-like factor 4 [68].

Hepatocellular carcinoma. The upregulated exosomal lncRNA HMMR-AS1 could boost M2 polarization via the miR-147a/ARID3A axis in hepatocellular carcinoma cells (HCC), thus leading to disease progression and poor prognosis [69]. In addition, the overexpression of lncRNA TUC399 can be encapsulated by HCC-derived exosomes and then taken up by a monocytic cell line THP-1 cells. This process benefits the polarization of THP-1 cells towards the M2 phenotype and diminishes its phagocytosis and pro-inflammatory cytokine production [70]. 

Lung cancer. Exosomal lncRNA SNHG7 secreted from lung adenocarcinoma (LUAD) cells is transferred to macrophages, which activates the phosphatidylinositol 3-kinase (PI3K)/AKT pathway and induces the phosphatase and tensin homolog (PTEN) degradation by recruiting cullin 4A, thus accelerating M2 polarization. The polarization of M2 macrophage can increase the docetaxel resistance in LUAD [71]. In non-small cell lung cancer (NSCLC), exosomal lncRNA SOX2 overlapping transcript (SOX2-OT) secreted from NSCLC cells boosts M2 polarization, thus increasing EGFR-TKI resistance by targeting the miR-627-3p/SMADs pathway [72]. In addition, NSCLC cell-secreted exosomal lncRNA PCAT6 has been found to induce M2 polarization, favoring tumor growth by promoting tumor cell invasion and migration [73]. Exosomal LINC00313 derived from NSCLC cells has also been reported to induce M2 polarization by activating STAT6 and benefit tumor progression [74]. Silencing of exosomal lncRNA FGD5-AS1 secreted from NSCLC cells can suppress M2 polarization to reduce migration and invasion of lung cancer cells [75].

Other tumors. Exosome-carrying lncRNA TP73-AS1 derived from nasopharyngeal carcinoma (NPC) can be transported to macrophages, which leads to M2 polarization and increased macrophage migration and tube formation [76]. In addition, LncRNA BCRT1 can be incorporated into exosomes to facilitate M2 polarization by targeting the miR-1303/PTBP3 axis in breast cancer, thus enhancing tumor cell migration and angiogenesis [77]. The overexpressed LncRNA ELFN1-AS1 in osteosarcoma (OS) cells could be transported via exosomes to macrophage, thereby boosting M2 polarization and OS progression [78]. Pancreatic cancer (PC) cell-derived exosomal FGD5-AS1 has been found to induce M2 polarization, thus accelerating PC cell growth and invasion [79]. Exosomes containing lncARSR secreted from renal cell carcinoma (RCC) were found to promote M2 polarization by activating the STAT3 pathway, thus favoring RCC progression [80]. In clear cell renal cell carcinoma (ccRCC), exosomal AP000439.2 derived from the tumor cells can promote their migration and growth by inducing M2 polarization via activating the STAT3/NF-κB signaling pathway [81].

To sum up, the above results indicate that exosomal lncRNAs derived from tumors usually promote M2 polarization or the production of inflammatory cytokines to support tumor progression and drug resistance.

#### 2.1.2. The Role of Exosomal lncRNAs in NK Cells

NK cells are an important innate immune cell population, functionally killing tumor cells or producing cytokines to strengthen immune response [82]. Upregulated expression of lncRNA GAS5 in NK cells is found to enhance their cytotoxicity to different cancer cells [40,83]. CRC-cell secreted exosomal lncRNA SNHG10 can be transferred into NK cells to activate the inhibin subunit beta C (INHBC)-dependent TGF-β signaling pathway, thus inhibiting NK cell-mediated cytotoxicity [84]. These results indicate that exosomal lncRNAs can regulate the cytotoxic activity of NK cells, therefore, participate in the immune response to affect tumor progression.

#### 2.1.3. The Role of Exosomal lncRNAs in CD4^+^ and CD8^+^T Cells

The adaptive immune response is mediated by T and B effector lymphocytes. Based on the different surface receptors and antigen specificity, the effector T cells could be classified into CD4^+^T helper (Th) and CD8^+^cytotoxic T lymphocytes (CTL) cells [85]. Due to the different biological roles, CD4^+^T cells can be further divided into T helper (Th) 1 cells, Th2, Th9, Th22, Th17 cells, follicular T helper (Tfh), and regulatory T (Treg) cells [86].

Th17 cells have been reported to benefit tumor progression by producing interleukin 17 (IL-17) [87]. Sun et al. revealed that CRC-secreted exosomes containing lncRNA CRNDE-h could be transmitted to CD4^+^T cells to increase RORγt transcription and further suppress its interaction with E3 ubiquitin ligase Itch to facilitate Th17 cells differentiation [88]. CRC-derived exosomes loaded with lncRNA KCNQ1OT1 can restrain the anticancer effect of CD8^+^T cells, thus facilitating CRC progression [89]. Collectively, tumor-secreted exosomal lncRNAs can promote Th17 cell differentiation or inhibit the function of normal CD8^+^T cells, thus contributing to tumorigenesis.

#### 2.1.4. The Role of Exosomal lncRNAs in Tregs

Tregs, a specialized subset of T cells, can be recruited to TME to construct an immunosuppressive environment [90]. Ni et al. found that CD73^+^γδ1 Tregs were the primary ones exerting the immunosuppressive function in the progression of breast cancer. Mechanistically, breast tumor cell-secreted lncRNA SNHG16 shuttles through exosomes to boost CD73 expression in γδ1 Tregs and activate the TGF-β1/SMAD5 pathway [91]. Furthermore, Wang et al. reported that exosomal lncRNA RP11-323N12.5 could be delivered to tumor-infiltrating leukocytes, enhancing Treg differentiation, and thus contributing to immunosuppression and tumor growth of GC through the YAP1/c-MYC axis [92]. To sum up, tumor-derived exosomal lncRNA can trigger Tregs differentiation to induce immunosuppression in TME.

#### 2.1.5. The Role of Exosomal lncRNAs in Bregs

Regulatory B cells (Bregs) are a special subpopulation of B lymphocytes that can inhibit the inflammatory response and hold immunological tolerance [93]. A few studies have demonstrated that Bregs can secrete IL-10 and TGF-β to restrain the immune response against tumors, thus beneficial to carcinogenesis [94,95,96]. A previous study has shown that ESCC-derived exosomes can restrict the expansion of B cells and promote the differentiation of Interleukin-10^+^ Bregs conversely. Further bioinformatic analysis has confirmed the significantly differentially expressed lncRNA profiles in ESCC-Exo compared with normal exosomes [97]. This result reveals that tumor-derived exosomal lncRNAs may promote Breg generation to escape immune surveillance.

### 2.2. Immune Cell-Derived Exosomal lncRNAs

In addition, exosomal lncRNAs from immune cells could be delivered into tumor cells to affect tumor progression and treatment efficacy. The underlying roles of macrophages- or CD8^+^T cell-derived exosomal lncRNAs in immune regulation and cancer pathogenesis are discussed as follows.

Macrophage-secreted exosomes. TAMs are one of the most plentiful immune cells infiltrated into TME [98]. TAMs can secrete exosomes containing lncRNAs to regulate tumor cell metabolism and survival. For example, TAM-secreted exosomes containing HIF-1α-stabilizing long noncoding RNA (HISLA) enhanced the aerobic glycolysis of breast cancer cells, further inhibiting cell apoptosis and promoting chemoresistance [99]. Furthermore, exosomal lncMMPA derived from TAMs is also found to facilitate M2 polarization and augment tumor’ aerobic glycolysis to promote HCC proliferation [100]. TAM-secreted exosomes with highly-expressed LIFR-AS1 can sink miR-29a to upregulate NFIA, thus supporting cell proliferation and invasion and suppressing apoptosis of OS cells [101]. However, the overexpression of recombination signal binding protein for immunoglobulin kappa J region in macrophage can secrete lncRNA LBX1-AS1-loaded exosomes to repress the proliferation and invasion of oral squamous cell carcinoma (OSCC) cells [102]. These studies indicated that the beneficial or harmful effects on tumors depend on the characteristics of macrophages.

M2-secreted exosomes. The lncRNAs encapsulated by M2-secreted exosomes have been proven to participate in tumor growth regulation and immune response by secreting cytokines like IL-10 and TGF-β. For instance, M2 macrophage-secreted exosomes (M2-Exo) transferred lncRNA AFAP1-AS1 at a high expression level to EC cells, which boosted the lung metastasis of cancer cells [103]. Hepatitis B virus (HBV)-related HCC can produce HBeAg to facilitate the expression of lncRNA MAPKAPK5_AS1 (MAAS) in M2 macrophages, thus transmitted by M2-Exo to HBV^+^HCC cells to promote their proliferation [104]. Upregulated lncRNA CRNDE in M2-Exo can be delivered to cisplatin-treated GC cells to promote their proliferation and enhance the resistance to cisplatin [105]. M2-Exo-loaded lncRNA AGAP2 antisense RNA 1 (AGAP2-AS1) can promote the radio resistance of lung cancer cells by negatively regulating miR-296 [106]. LINC00273-containing exosomes secreted from the M2 subtype are found to improve the invasion, migration, and metastasis of LUAD cells by regulating the Hippo/Yes-associated transcriptional regulator pathway [107]. The M2-Exo containing the highly expressed lncRNA SBF2-AS1 can significantly upregulate the expression of X-linked inhibitor of apoptosis protein by competitively binding to miR-122-5p, thus contributing to PC progression [108]. M2-secreted exosomal lncRNA H19 can facilitate the autophagy process of bladder cancer (BC) through stabilizing Unc-51-like autophagy activating kinase 1 (ULK1) [109]. Taken together, the delivery of aberrant lncRNA in M2-secreted exosomes often benefits tumor progression or enhances the chemoresistance or radio resistance of tumor cells.

M1-secreted exosomes. M1 macrophages have been reported to transfer exosomal lncRNA to tumor cells to affect tumor progression. M1-derived exosomes (M1-Exo) loaded with lncRNA HOTTIP can promote the apoptosis of head and neck squamous cell carcinoma (HNSCC) cells and repress the progression. The mechanistic investigation has unveiled that lncRNA HOTTIP upregulates the TLR5/NF-κB signaling pathway by binding to miR-19a-3p and miR-19b-3p [110]. Therefore, certain lncRNAs in M1-Exo may inhibit the progression of tumors and serve as a potential therapeutic target. 

CD8^+^T cell-secreted exosomes. As previously mentioned, tumor-secreted exosomal lncRNAs can interfere with the cytotoxic ability of CD8^+^T cells to tumor cells. Furthermore, normal CD8^+^T cells could be inhibited by their exhausted population. Accompanied by low cytotoxic ability, the exhausted CD8^+^T cells have been shown to deliver self-generated exosomes to normal CD8^+^T cells, thus impairing their proliferation and activity [111].

## 3. Exosomal lncRNA-Based Cancer Treatment

Emerging studies have demonstrated the significant linkage between exosomal lncRNAs and immune regulation in cancers. Aberrant exosome-associated lncRNAs could affect the sensitivity to therapeutic strategies by regulating the infiltration and function of immune cells in cancers (Table 1). Based on the immune-regulatory mechanism of exosomal lncRNAs in cancers, increasing studies tend to explore the corresponding therapeutic strategies targeting exosomal lncRNAs. 

Given the side-effect and resistance of traditional therapy, such as chemotherapy and radiotherapy, immunotherapy is considered a promising option to fight cancer [112,113]. The application of immunotherapy can effectively identify and eliminate tumor cells by enhancing the natural anticancer capacity of the host’s immune cells. With the advance in research of immunotherapy in past decades, vaccines, chimeric antigen receptor T-cell (CAR-T) therapy, immune checkpoint inhibitors, and combinatorial therapies have emerged as potential immunotherapies against cancer [114,115,116,117]. One of the most prospective immunotherapy strategies is the immune checkpoint inhibitors. In physiological conditions, immune checkpoints, such as PD-1 and CTLA-4, are expressed on the surface of immune cells, maintaining immunological tolerance and avoiding autoimmunity appearance [118]. Tumor cells exploit their surface ligands to combine with corresponding immune checkpoint receptors, thus suppressing the anti-tumor immune response of immune cells [119]. 

Emerging evidence has revealed that exosomal lncRNAs can regulate the ligand-receptor interplay of PD-L1/PD-1 to escape the immune attack of immune cells in various cancers. For instance, Xing et al. reported that the downregulation of lncRNA XIST boosted the production of tumor-secreted exosomal miR-503, which enhanced the expression of PD-L1 on M2 macrophages to suppress T proliferation in breast cancer [62]. Exosomes secreted by ESCC cells can induce the high expression of PD-1 on Bregs by activating the TLR4/MAPK signaling pathways, thus promoting B cell-mediated immunosuppression [97]. CRC-secreted exosomes can deliver lncRNA KCNQ1OT1 to themselves, thus inhibiting the ubiquitination of PD-L1 from escaping the CD8^+^T cell-mediated immune surveillance [89]. Exosomal lncRNA PCED1B-AS1 derived from HCC can enhance the expression of PD-L1 and PD-L2 on HCC cells and function as a suppressor to T cells and macrophages [120]. These results demonstrate that exosomal lncRNAs can promote the tumor’s escape from immune surveillance by enhancing the immunosuppression of M2 and Bregs or upregulating the expression of PD-L1 and PD-L2 on tumor cells. Therefore, the application of immune checkpoint inhibitors may block the exosomal lncRNA-mediated receptor-ligand interaction between tumor and immune cells, thus enhancing anti-tumor immunity [121]. 

A recent study also revealed that lncRNA NORAD knockdown induces the secretion of exosomes containing miR-199a-5p to suppress the ubiquitination of PD-L1 in an ESCC mouse model, resulting in enhanced efficacy of anti-PD-1 in combination with radiation [122]. In addition, Li et al. also identified that B7-H3 and VSIR might be novel checkpoints for their upregulated expression in GC patients, affecting the efficacy of immunotherapy [123]. These results indicate that the application of immune checkpoint inhibitors or antibodies aiming to interfere with exosomal lncRNA-mediated checkpoint interaction benefits the normal function of immune cells, thereby improving the efficacy of cancer treatment.

Given that exosomes carrying lncRNAs play the role of immune regulation in cancer, interventions of exosomal lncRNAs, such as siRNA, are also one of the potential therapeutic modalities. Characterized by double-stranded RNAs with 20 to 24 nucleotides, siRNA can inhibit mRNA transcription by inducing heterochromatin assembly and suppress mRNA translation or facilitate the degradation of mRNA or pre-mRNA to regulate gene expression [124]. Furthermore, siRNAs can be delivered and integrated into the genome of target cells, thus leading to post-transcriptional regulation of lncRNAs. Certain siRNAs complementarily combine with target lncRNAs to construct an RNA-induced silencing complex, thereby splicing and degrading the target lncRNA [125]. Accordingly, siRNAs can interfere with aberrant lncRNA expression, thus affecting tumor progression [126]. Chen et al. revealed that using siRNAs to knock down PCAT6 in NSCLC cells can suppress M2 polarization, thus inhibiting tumor growth [73]. Due to the good biocompatibility of exosomes, they are used as delivery mediators to protect siRNAs from endosome-mediated degradation. For instance, upregulated lncRNA DARS-AS1 was found to facilitate the tumorigenesis and metastasis of triple-negative breast cancer (TNBC), whereas DARS-AS1 siRNA-loaded exosomes inhibited the growth and metastasis of TNBC [127]. Guo et al. found that upregulated lncRNA H19 in M2-secreted exosomes promoted BC cell growth, whereas the application of M2-Exo-siRNA H19 inhibited BC progression [109]. These results indicate that exosomal siRNAs targeting exosomal lncRNAs may offer potential value in cancer therapy.

## 4. Conclusions 

**Figure 2:**
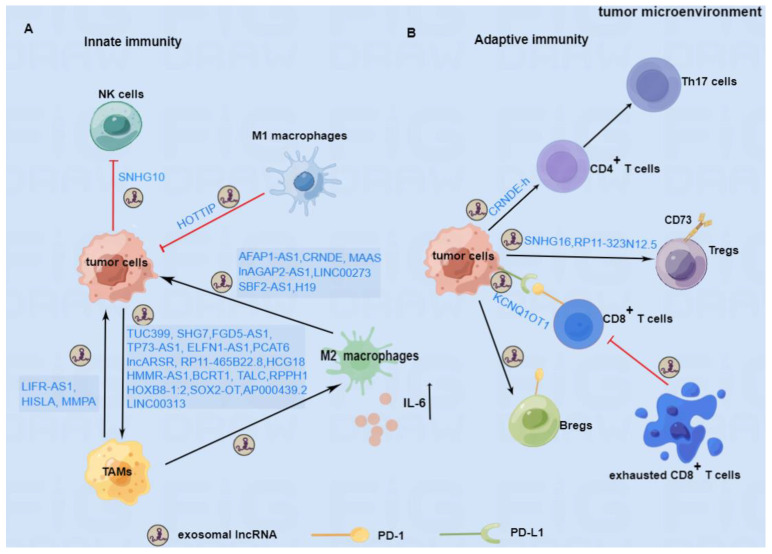
The immunomodulatory role of exosomal lncRNAs in TME. (**A**) In innate immunity, tumor cells can secret exosomal lncRNAs to boost the polarization of M2 macrophages or inhibit the killing function of NK cells. TAMs and M2 macrophage-secreted exosomal lncRNAs can support the progression of tumor cells, whereas M1 macrophage-derived exosomal lncRNA may inhibit the progression of tumors. (**B**) In adaptive immunity, tumor cells can secrete exosomal lncRNAs to promote the differentiation of Th17 cells, Tregs, and Bregs and the PD-1 expression on the surface of Bregs, which are beneficial to the progression of tumors. In addition, tumor cells can also secrete exosomes containing lncRNAs to maintain the PD-L1 expression on their surface, thus restraining the biological function of CD8^+^T cells. Furthermore, exhausted CD8^+^T cells can also produce certain exosomal lncRNA to impair the ability of normal CD8^+^T cells. LncRNAs involved in the mechanism of immune regulation are depicted above.

In this review, we summarized the emerging studies about the role of exosomal lncRNAs in the immune response of cancer and proposed possible therapeutic strategies targeting exosomal lncRNAs. As shown in Figure 2, exosomal lncRNAs can regulate macrophage polarization, inhibit the function of NK cells, and promote the secretion of inflammatory factors to participate in the tumor’s innate immune responses. Furthermore, exosomal lncRNAs can inhibit the activity of CD8^+^ T cells and facilitate the differentiation of Th17 cells, Tregs, and Bregs to regulate the adaptive immune response in cancer. Based on the immunomodulatory mechanism of exosomal lncRNAs, blockade of immune checkpoints represented by the PD-1/PD-L1 antibodies or inhibitors to stimulate the immune response, and exosomes carrying siRNAs to interfere with exosomal lncRNAs have made promising progress in cancer therapy. However, most results are still at the stage of basic investigation or preclinical tests. To date, effectively targeting exosomal lncRNAs to achieve better outcomes in cancer remains a challenge. The research needs to be accelerated to solve the existing problems and obtain clinical benefits.

## Figures and Tables

**Table 1:** Summary of immunoregulation role of exosomal lncRNAs in cancer.

Exosomal lncRNAs	Exosome Source	Expression	Biological Function	Cancer Type	Ref
**BCRT1**	Breast cancer cell	Up	Promoting M2 polarization and enhancing tumor cell migration and angiogenesis	Breast cancer	[77]
**TALC**	Glioblastoma cell	Unknown	Manipulating the process of M2 polarization and reducing tumor sensitivity to temozolomide	Glioblastoma	[63]
**RPPH1**	CRC cell	Up	Provoking the M2 polarization, thus causing CRC cells’ metastasis and proliferation	CRC	[65]
**HOXB8-1:2**	neuroendocrine differentiated CRC	Up	Promoting M2 polarization, thus boosting the progression and metastasis of CRC cells	CRC	[66]
**RP11-465B22.8**	EC cell	Up	Promoting M2 polarization and subsequent EC cells migration and invasion	EC	[67]
**HCG18**	GC cell	Up	Promoting M2 polarization	GC	[68]
**HMMR-AS1**	HCC cell	Up	Boosting M2 polarization and accelerating the progression of HCC	HCC	[69]
**TUC399**	HCC cell	Up	Regulating cytokine secretion of macrophage and M2 polarization	HCC	[70]
**SNHG7**	LUAD cell	Up	Accelerating M2 polarization and increasing the resistance of docetaxel in LUAD	Lung cancer	[71]
**SOX2-OT**	NSCLC cell	Up	Boosting the M2 polarization, thus increasing EGFR-TKI resistance	NSCLC	[72]
**PCAT6**	NSCLC cell	Up	Facilitating tumor cell growth by inducing M2 polarization	NSCLC	[73]
**LINC00313**	NSCLC cell	Up	Facilitating M2 polarization and tumor progression	NSCLC	[74]
**FGD5-AS1**	NSCLC cell	Up	Promoting M2 polarization, thus increasing migration and invasion of NSCLC cell	NSCLC	[75]
**TP73-AS1**	NPC cell	Up	Promoting M2 polarization and enhancing the motility and tube formation of macrophages	NPC	[76]
**ELFN1-AS1**	OS cell	Up	Boosting M2 polarization, thus promoting the tumorigenesis of OS	OS	[78]
**FGD5-AS1**	PC cells	Up	Boosting M2 polarization, thus stimulating tumor cells progression	PC	[79]
**LncARSR**	RCC cell	Up	Promoting the progression of RCC by inducing macrophage polarization	RCC	[80]
**AP000439.2**	ccRCC cell	Unkown	Promoting the progression of RCC by inducing M2 polarization	ccRCC	[81]
**AFAP1-AS1**	M2 macrophage	Up	Promoting the migration, invasion, and lung metastasis of EC cells	EC	[103]
**CRNDE**	M2 macrophage	Up	Enhancing cisplatin resistance of GC cells	GC	[105]
**MAAS**	M2 macrophage	Up	Boosting the proliferation of HBV^+^HCC cells	HCC	[104]
**AGAP2-AS1**	M2 macrophage	Up	Promoting the radioresistant of lung cancer cells	Lung cancer	[106]
**LINC00273**	M2 macrophage	Up	Promoting LUAD invasion and migration and metastasis	Lung cancer	[107]
**HISLA**	TAM	Up	Enhancing the aerobic glycolysis and apoptotic resistance of breast cancer cells	Breast cancer	[99]
**LncMMPA**	TAM	Up	Boosting M2 polarization and aerobic glycolysis to support tumor cells proliferation	HCC	[100]
**LIFR-AS1**	TAM	Up	Boosting OS cells proliferation, invasion, and inhibiting their apoptosis	OS	[101]
**SBF2-AS1**	M2 macrophage	Up	Promoting the progression of PC	PC	[108]
**H19**	M2 macrophage	Up	Promoting the autophagy of BC cells	BC	[109]
**LBX1-AS1**	Macrophage	Up	Repressing the proliferation and invasion of OSCC cells	OSCC	[102]
**HOTTIP**	M1 macrophage	Up	Repressing the HNSCC’s progression and promoting the apoptosis of HNSCC cells	HNSCC	[110]
**SNHG10**	CRC cell	Up	Inhibiting the function of NK cells	CRC	[84]
**CRNDE-h**	CRC cell	Up	Facilitating the Th17 cells differentiation	CRC	[88]
**KCNQ1OT1**	CRC cell	Up	Inhibiting the function of CD8^+^T cells	CRC	[89]
**SNHG16**	Breast cancer cell	Unknown	Boosting CD73 expression in γδ1 Treg cells to exert immunosuppression	Breast cancer	[91]
**RP11-323N12.5**	GC cell	Up	Inducing Treg differentiation and facilitating GC cells growth and immunosuppression	GC	[92]
